# Supplementary material for: Orai1 is required for Ca2+-dependent plasma membrane repair and mechanoadaptation
Source: bioRxiv. 2026 May 15:2026.05.13.724989. Preprint. [Version 2] doi: 10.64898/2026.05.13.724989 (PMC13193018; doi:10.64898/2026.05.13.724989)
Supplement: 1 [file NIHPP2026.05.13.724989V2-supplement-1.pdf]

Fig S1

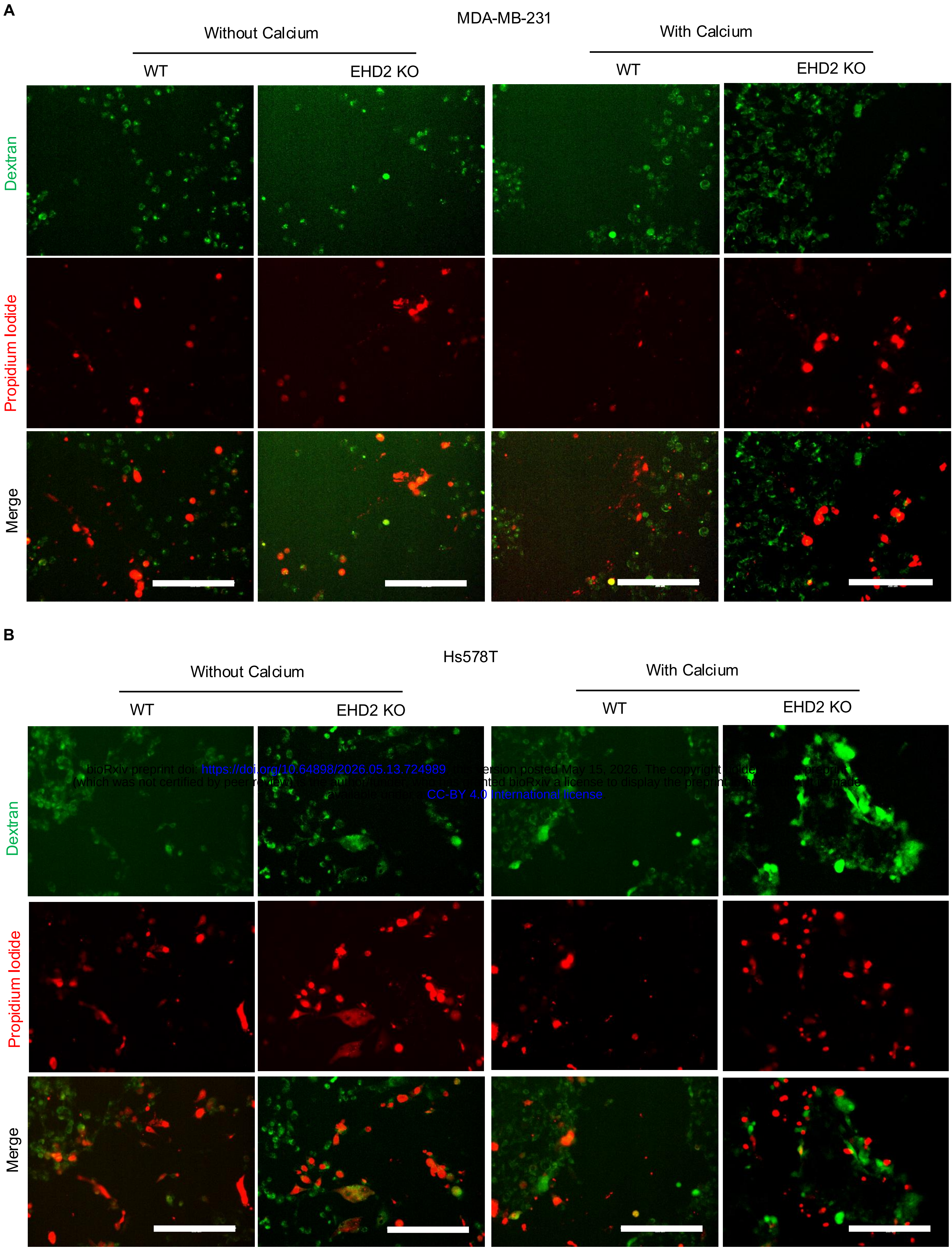

bioRxiv preprint doi: https://doi.org/10.64898/2026.05.13.724989; this version posted May 15, 2026. The copyright holder for this preprint (which was not certified by peer review) is the author/funder, who has granted bioRxiv a license to display the preprint in perpetuity. It is made available under a CC-BY 4.0 International license.

Fig S2

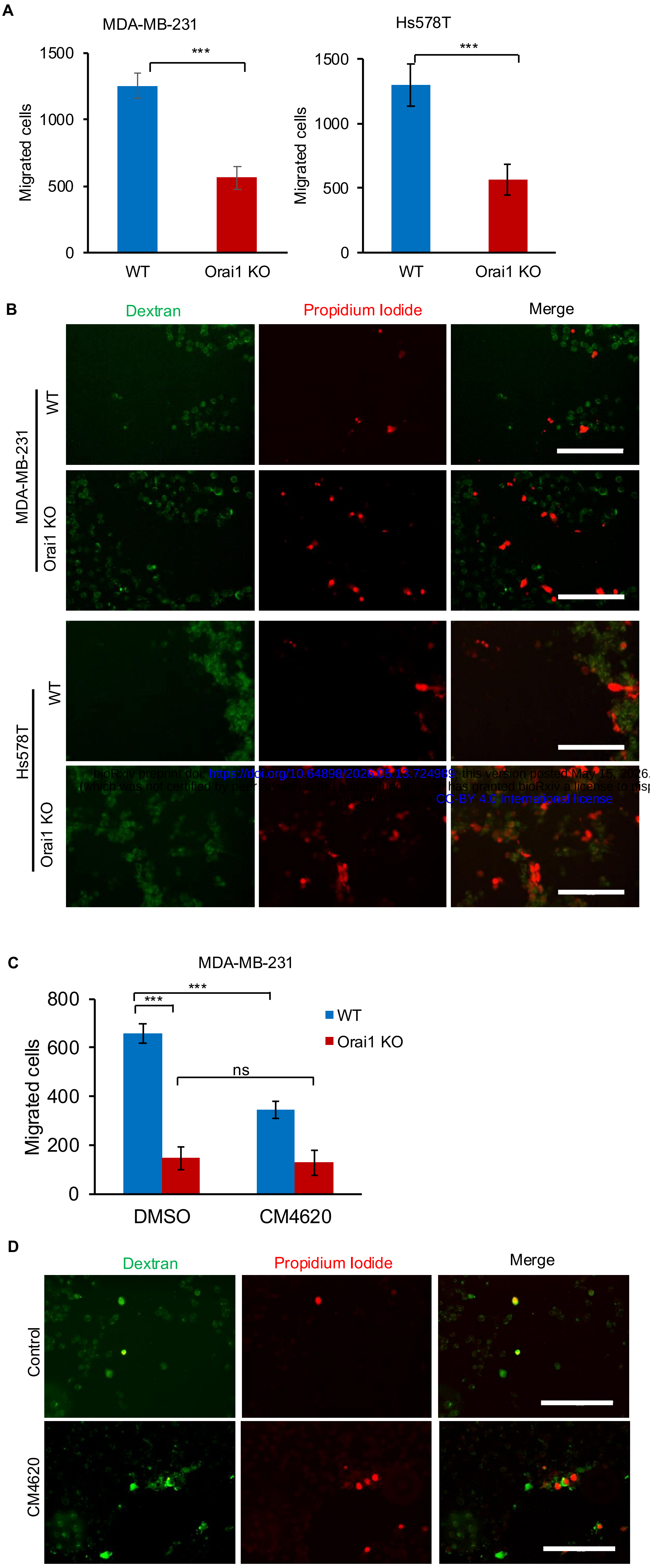

bioRxiv preprint doi: https://doi.org/10.64898/2026.05.13.724989; this version posted May 15, 2026. The copyright holder for this preprint (which was not certified by peer review) is the author/funder, who has granted bioRxiv a license to display the preprint in perpetuity. It is made available under a CC-BY 4.0 International license.

Fig S3

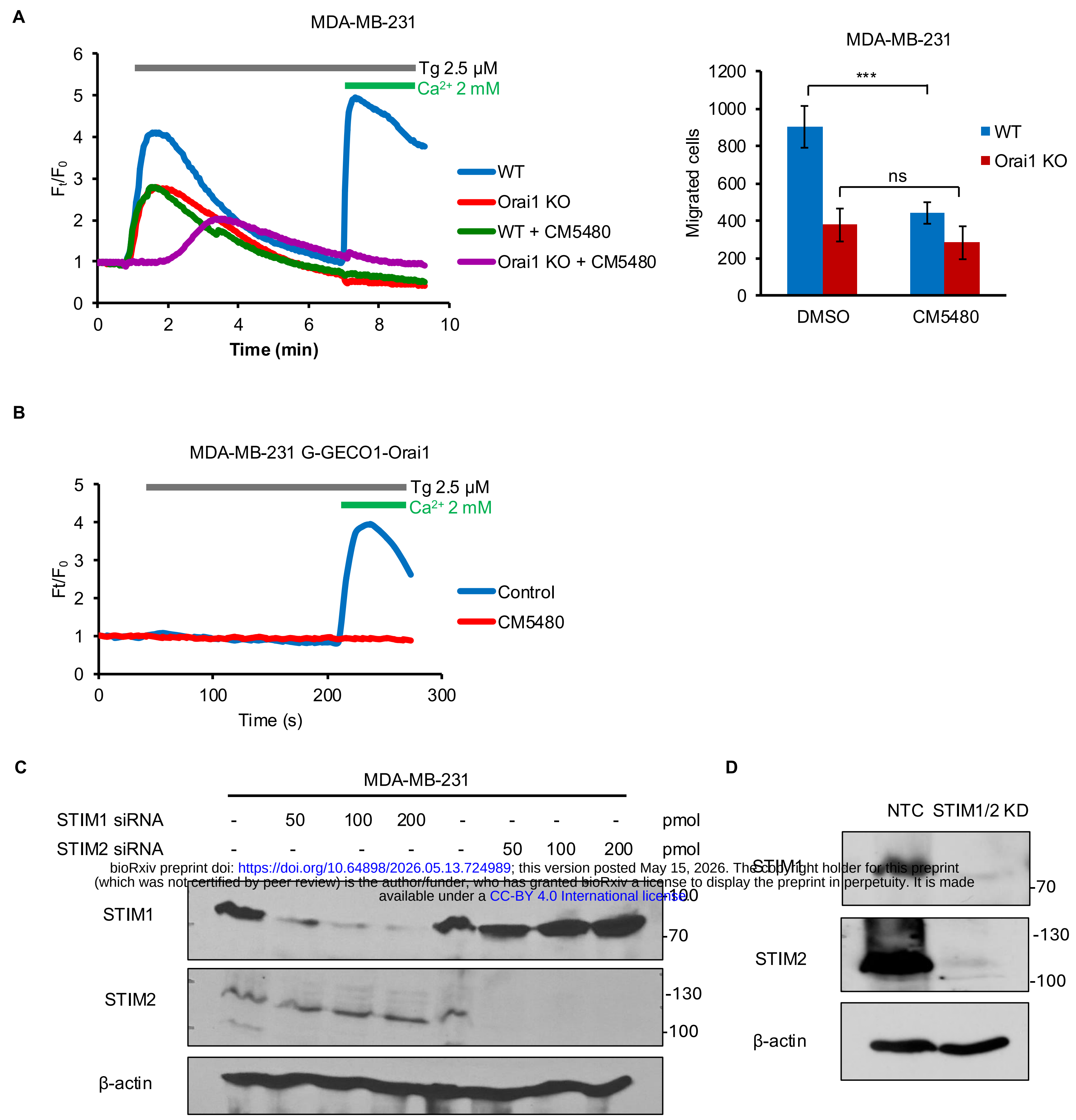

bioRxiv preprint doi: https://doi.org/10.64898/2026.05.13.724989; this version posted May 15, 2026. The copyright holder for this preprint (which was not certified by peer review) is the author/funder, who has granted bioRxiv a license to display the preprint in perpetuity. It is made available under a CC-BY 4.0 International license.

Fig S4

A

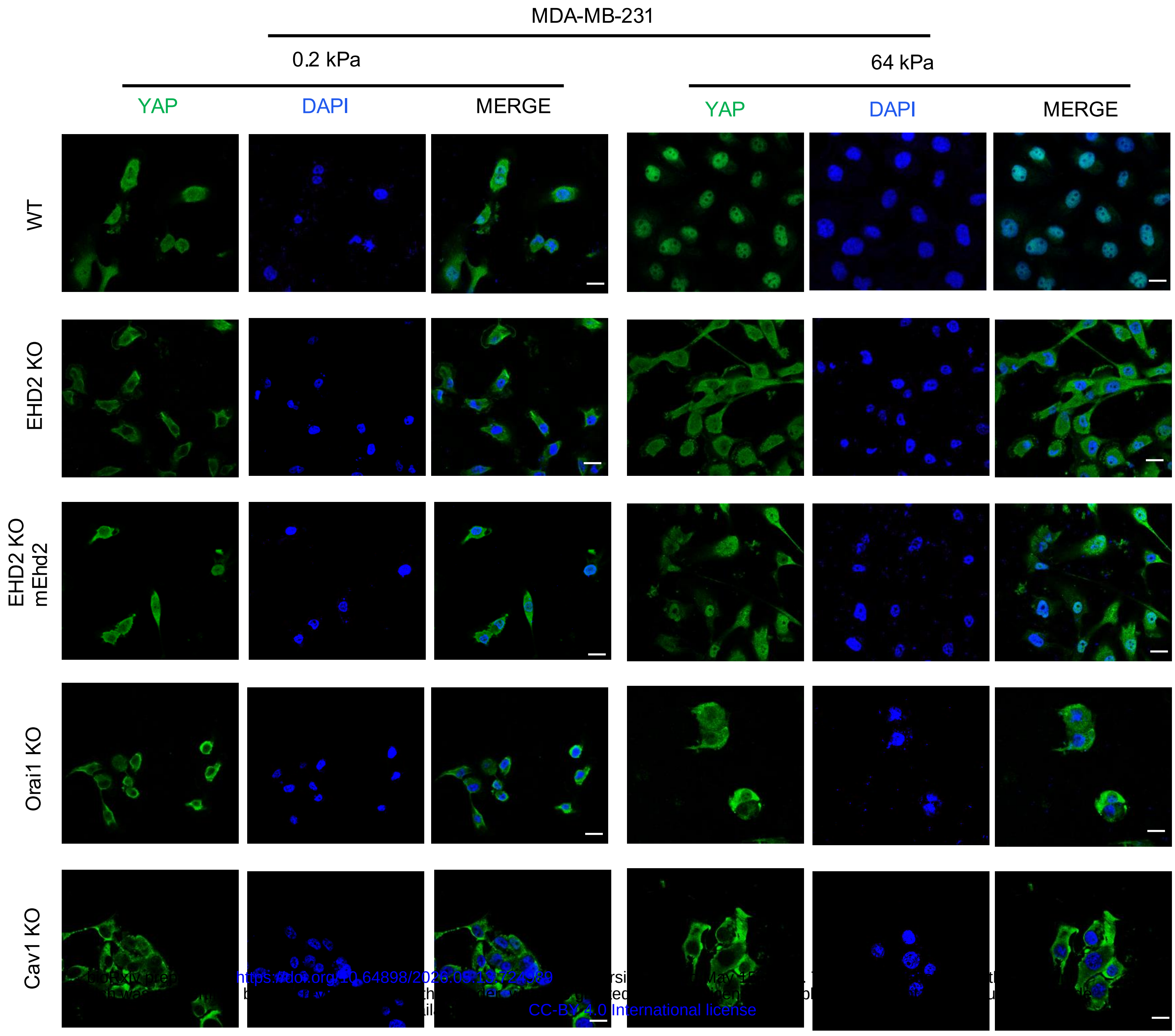
MDA-MB-231
0.2 kPa
64 kPa
YAP
DAPI
MERGE
YAP
DAPI
MERGE
WT
EHD2 KO
EHD2 KO mEhd2
Orai1 KO
Cav1 KO

B

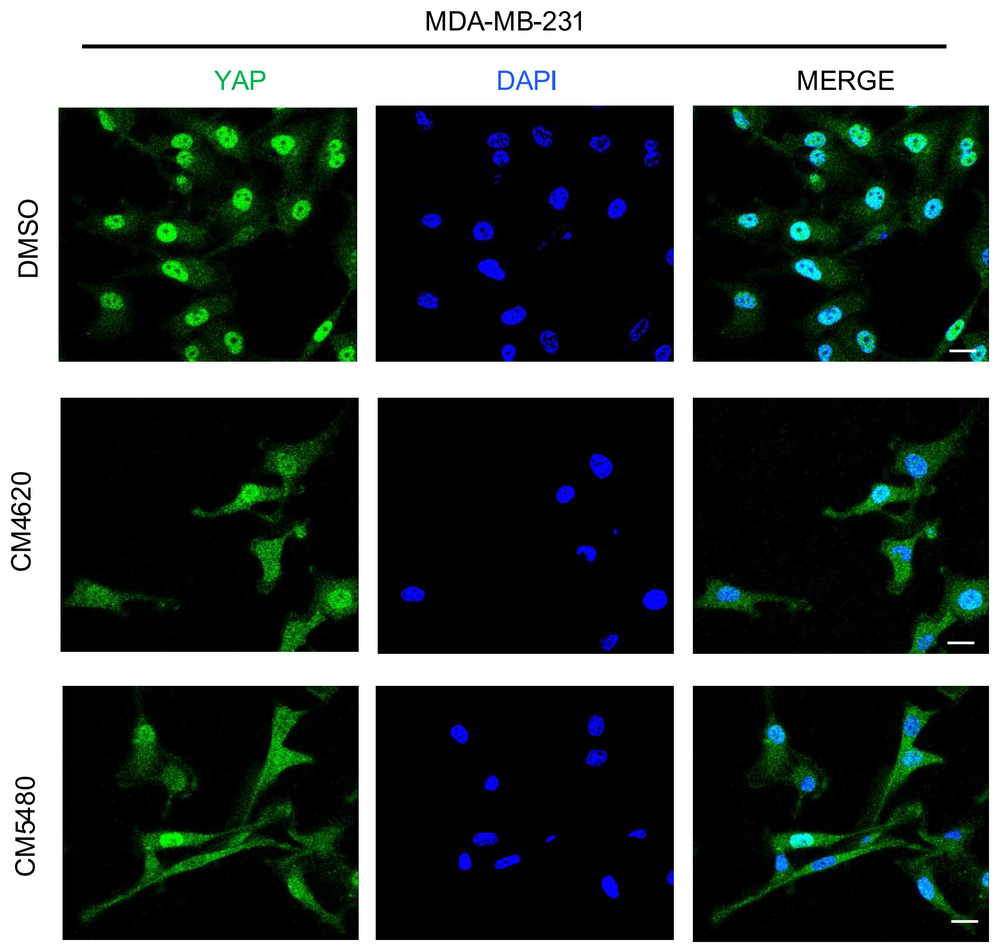
MDA-MB-231
YAP
DAPI
MERGE
DMSO
CM4620
CM5480

Fig S5

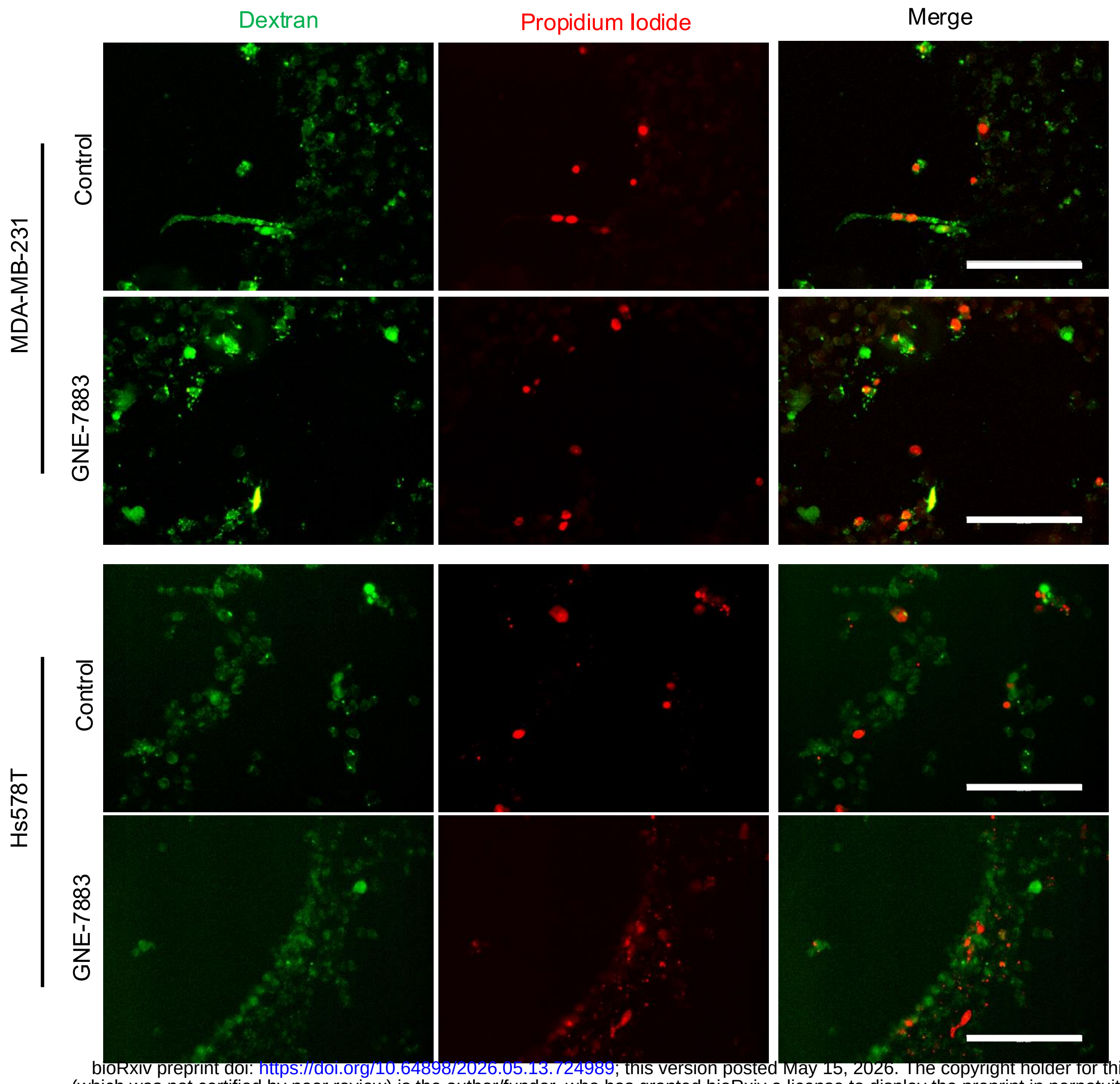

bioRxiv preprint doi: https://doi.org/10.64898/2026.05.13.724989; this version posted May 15, 2026. The copyright holder for this preprint (which was not certified by peer review) is the author/funder, who has granted bioRxiv a license to display the preprint in perpetuity. It is made available under a CC-BY 4.0 International license.
